# Trastuzumab-functionalized bionic pyrotinib liposomes for targeted therapy of HER2-positive breast cancer

**DOI:** 10.1186/s13058-024-01853-2

**Published:** 2024-06-12

**Authors:** Jiaqun Du, Xiaobang Liu, Junpeng Sun, Qian Wu, Yu Hu, Huan Shi, Li Zheng, Ying Liu, Chao Wu, Yu Gao

**Affiliations:** 1 Department of Medical Oncology, The First Affiliated Hospital of Jinzhou Medical University, No. 2, the Fifth Section of Renmin Street, Guta District, Jinzhou, 121001 Liaoning Province China; 2 Pharmacy School, Jinzhou Medical University, 40 Songpo Road, Linghe, Jinzhou, 121001 Liaoning China

**Keywords:** Pyrotinib, Trastuzumab, SK-BR-3 cell membranes, Liposome, HER2-positive breast cancer, Targeted therapy

## Abstract

In this study, we prepared a bionic nanosystem of trastuzumab-functionalized SK-BR-3 cell membrane hybrid liposome-coated pyrotinib (Ptb-M-Lip-Her) for the treatment of HER2-positive breast cancer. Transmission electron microscopy, dynamic light scattering, polyacrylamide gel electrophoresis (SDS-PAGE) and western blotting were used to verify the successful preparation of Ptb-M-Lip-Her. In vitro drug release experiments proved that Ptb-M-Lip-Her had a sustained release effect. Cell uptake experiments and in vivo imaging experiments proved that Ptb-M-Lip-Her had good targeting ability to homologous tumor cells (SK-BR-3). The results of cell experiments such as MTT, flow cytometry, immunofluorescence staining and in vivo antitumor experiments showed that Ptb-M-Lip-Her could significantly promote apoptosis and inhibit the proliferation of SK-BR-3 cells. These results clearly indicated that Ptb-M-Lip-Her may be a promising biomimetic nanosystem for targeted therapy of HER2-positive breast cancer.

**Supplementary Information:**

The online version contains supplementary material available at 10.1186/s13058-024-01853-2.

## Introduction

Breast cancer is a serious threat to global women's health, and human epidermal growth factor receptor 2 (HER2)-positive breast cancer accounts for approximately 15–20% [1–3]. Compared with other types, HER2-positive breast cancer is a subtype of breast cancer with strong aggressiveness and a high degree of malignancy, featuring poor prognosis, easy metastasis and a high risk of death [4, 5]. At present, the clinical treatment of HER2-positive breast cancer is mainly chemotherapy, except for surgery [6, 7]. Clinically, chemotherapy drugs often cause toxic side effects, drug resistance and other problems, which seriously affect the therapeutic effect [8–10]. Commonly used chemotherapy drugs include paclitaxel, doxorubicin, monoclonal antibodies (trastuzumab, etc.), pyrotinib, etc. [11–13]. Among them, pyrotinib, as a novel small molecule intracellular targeted drug, can covalently bind to the adenosine triphosphate binding sites of HER family proteins (HER1, HER2 and HER4) in the intracellular kinase region and can also prevent the formation of homodimers/heterodimers of HER family proteins, thereby inhibiting its own phosphorylation and the activation of downstream signaling pathways, thus inhibiting tumor growth [14, 15]. At present, the double-targeted combination of a monoclonal antibody drug (Herceptin) and pyrotinib is often used in the clinical treatment of advanced HER2-positive breast cancer with good effects [16–18]. However, currently commercially available pyrotinib preparations are administered orally and are often associated with adverse reactions such as neutropenia and diarrhea [19, 20]. Herceptin is often accompanied by drug resistance and other problems [21, 22]. How to use effective technical means to optimize the treatment strategy and overcome the above problems is of great significance for the treatment of HER2-positive breast cancer.

In recent years, nanodrug delivery systems have provided new ideas for cancer therapy, such as liposomes [23], polymer nanoparticles [24] and polymer micelles [25], which can be surface modified (ligands or antibodies, etc.) and widely loaded with drugs to achieve high targeting and bioavailability [26–28]. Among them, liposomes, as a mature preparation technology, have been widely used, with the characteristics of targeting, slow release, reduced drug toxicity and improved stability [29, 30]. With the continuous development of biomaterials technology, biomimetic lipid vesicles, such as cell membrane vesicles, exosomes, and apoptotic body membrane vesicles, have attracted extensive attention from researchers [31–33]. Their basic structure is the same as that of liposomes, which are composed of a phospholipid bilayer. Therefore, biomimetic lipid vesicles not only have homologous characteristics but also have the characteristics of liposomes. However, the preparation of such biomimetic materials is complicated, the yield is low, and the cost is high [34, 35]. Therefore, we designed a cell membrane fusion liposome as a drug carrier based on the similar principle of the cell membrane and liposome phospholipid bilayer. In this way, a small amount of cell membrane could be fused with liposomes so that the liposomes could have homology tropism, which not only realized bionics but also simplified the process and saved costs. Inspired by the clinical combination therapy regimen of Herceptin and pyrotinib, we bonded Herceptin with the HER2-positive cancer cell membrane through amide bonds and then fused it with pyrotinib liposomes to obtain trastuzumab-functionalized bionic pyrotinib liposomes. This design, on the one hand, achieves the macromolecule trastuzumab and HER2 extracellular domain binding; on the other hand, the small molecule pyrotinib can achieve accumulation in tumor tissue through cell internalization or diffusion and combine with the intracellular domain of the HER family so that the two can realize the "inner and outer combination" and achieve the extracellular and intracellular synergistic anti-HER2 tumor effect. At the same time, the homologous targeting of the SK-BR-3 cell membrane further improves the ability of drug aggregation in tumor sites. Therefore, this study verifies the targeting and antitumor ability of Ptb-M-Lip-Her through in vitro cell experiments and in vivo animal experiments, providing a new idea for the clinical treatment of HER2-positive breast cancer.

## Materials and methods

### Materials

Pyrotinib was ordered from Jiangsu Hengrui Pharmaceutical Co., Ltd (Jiangsu, China) with > 99% purity. Herceptin was purchased from Roche Pharmaceuticals Co., Ltd. (Shanghai, China). N-hydroxysuccinimide (NHS), 1-ethyl-3-[3-dimethylaminopropyl] carbodiimide hydrochloride (EDC), dimethyl sulfoxide (DMSO), sodium dodecyl sulfate (SDS), 98% soy phosphatidylcholine, 99% cholesterol, chloroform, ethanol, methanol, and paraformaldehyde were purchased from Aladdin Chemical Reagent Co., Ltd. (Shanghai, China). Thiazolyl blue tetrazolium bromide (MTT), 7-AAD, Annexin V-FITC apoptosis detection kit, trypsin, bovine serum albumin, and Triton X-100 were supplied by Nanjing Jiancheng Bioengineering Institute (Nanjing, China). SK-BR-3 cell lines were obtained from Keygen Biotech (Jiangsu, China). McCoy's 5A medium and fetal bovine serum (FBS) were purchased from Beijing Dingguo Changsheng Biotech Co., Ltd. (Beijing, China). Phenylmethanesulfonyl fluoride (PMSF), a mitochondrial membrane potential assay kit (JC-1), and calcein-AM/PI were provided by Beyotime Biotechnology Co., Ltd. (Shanghai, China). Anti-Bcl-2, anti-Bax, and anti-Caspase-3 antibodies were obtained from Abcam (Cambridge, UK). N-cadherin, galectin-3, EpCAM, ErbB-2, and EGFR were purchased from Wanleibio (Liaoning, China).

### Preparation of Ptb-M-Lip-Her

#### SK-BR-3 cell membrane derivation

SK-BR-3 cells were cultured in McCoy's 5A medium containing 10% FBS (37 °C, 5% carbon dioxide). When SK-BR-3 cells were overgrown, they were digested with trypsin and collected by centrifugation at 1000 rpm/min. The cells were washed three times with precooled PBS and then treated with 0.2% PBS for 24 h. The cell suspension was centrifuged at 1250 rpm for 15 min, and the supernatant was discarded. NaHCO3 (1 mM), EDTA (0.2 mM) and PMSF (100 mM) were added to the precipitate, and the cell suspension was transferred to a homogenizer and ground 30 times. The supernatant was collected after the suspension was centrifuged at 1250 rpm for 30 min. The supernatant from both collections was centrifuged at 12,000 rpm for 20 min, and the supernatant was discarded. The remaining precipitate was the SK-BR-3 cell membrane, which was stored at − 20 °C for further use.

#### Preparation of Ptb-M-Lip-Her and related particles

NHS and EDC (5 mg/mL) were added to the cell membrane to activate carboxyl groups on the cell membrane. Then, 1 mg of Herceptin was dissolved in 1 mL of PBS and mixed with the cell membrane after activation of the carboxyl group. The mixture was incubated at room temperature for 6 h to obtain M-Her. Then, the sample was centrifuged at 12,000×*g* for 30 min, and the supernatant was collected. The content of Herceptin in the supernatant was determined with a BCA kit. The grafting amount of Herceptin was calculated using the following formula.

$${\text{P}}_{{{\text{ac}}}} = {\text{P}}_{{\text{i}}} - {\text{P}}_{{{\text{nc}}}}$$

P_ac_ is the amount of connected HCT (mg), P_i_ is the initial amount of HCT (mg), and P_nc_ is the amount of nonconnected HCT (mg) in the supernatant.

Soy phosphatidylcholine and cholesterol were added to the round-bottomed flask at a mass ratio of 3:1, and chloroform was added to dissolve it fully [36]. Then, in a 37 °C constant temperature water bath, the chloroform was evaporated by rotary evaporation, and finally, the lipid membrane was formed. The lipid membrane was hydrated for 30 min with a saline solution containing Herceptin-modified cell membrane and pyrotinib for 30 min to preliminarily mix with lipids. The system was then ultrasonicated in an ice bath for 5 min at 10-s intervals and incubated at 37 °C for 30 min to achieve complete fusion of the cell membrane and lipid. Finally, the whole nanoparticle was successively extruded by the liposome extruder at 0.4 μm, 0.2 μm and 0.1 μm. Then, Ptb-M-Lip-Her was isolated by a dextran gel column. To prepare the drug-loaded liposomes without biomimetics modification (Ptb-Lip), the normal saline solution did not contain the SK-BR-3 cell membrane but only contained Ptb. To prepare FITC-labeled liposomes, the normal saline solution contained Ptb, SK-BR-3 cell membrane and FITC. The next steps in the preparation process were the same as those described above.

### Determination of drug loading

The prepared Ptb-M-Lip-Her was ultrasonicated with an ultrasonic probe at 200 W for 5 min at intervals of 10 s and then centrifuged at a rate of 80,000 r/min for 20 min. The supernatant was filtered through a 0.22 μm filter membrane, and the absorbance of Ptb was determined by ultraviolet spectrophotometry (UV-2000, languages, Franksville, WI).

$${\text{Encapsulation}}\,{\text{rate}}\% = ({\text{encapsulated}}\,{\text{drugs}}/{\text{total}}\,{\text{drug}}\,{\text{volume}}) \times 100\%$$

### Characterization

#### Morphology and characterization of nanomaterials

The morphologies of Lip and Ptb-M-Lip-Her were observed by transmission electron microscopy (TEM, Jem-1400 Flash, Japan). The particle size, zeta potential and stability of Lip, Ptb-M-Lip and Ptb-M-Lip-Her were measured using a laser diffraction particle size analyzer (Nano-ZS90, Malvern, Malvern, UK).

#### Membrane protein characterization

The membrane protein of the SK-BR-3 cell membrane was characterized by sodium dodecyl sulfate-polyacrylamide gel electrophoresis (SDS-PAGE), and the successful encapsulation of the SK-BR-3 cell membrane was verified. To put it simply, SK-BR-3 cell membrane, Ptb-M-Lip and Ptb-M-Lip were lysed by radioimmunoprecipitation assay (RIPA) lysis buffer (Dingguo, China). A bicinchoninic acid protein assay (BCA) kit was then used to assay the protein concentration. The sample was mixed with SDS-PAGE sample loading buffer (Dingguo, China) and heated at 100 °C for 5 min. Samples with the same amount of protein (10 μL/well) were then loaded on an 8% SDS-PAGE gel and electrophoresed for 2 h under constant pressure. The obtained gels were stained with Komas blue for 2 h and analyzed with Quantity One 1-D analysis software (Bio-Rad, Hercules, USA). Western blot analysis of SK-BR-3 cell membrane, Ptb-M-Lip and Ptb-M-Lip-Her surface proteins (ErbB-2, EGFR, EpCAM, N-cadherin, and Galectin-3) further verified the successful encapsulation of SK-BR-3 cell membrane.

### In vitro drug release study

The release properties of Ptb-M-Lip-Her in vitro were investigated using phosphate buffer solution as the release medium. Ptb-M-Lip-Her was placed in a dialysis bag (MWCO 3500). At the predetermined time, 3 mL of solution was taken and then injected into the same volume of PBS. The absorbance of Ptb was measured at 260 nm by an ultraviolet spectrophotometer (UV-2000, languages, Franksville, WI), and the cumulative release was calculated. The experiment was repeated three times, and the results were averaged and expressed as the standard deviation (± SD).

### In vitro cell assay

#### Cell culture

SK-BR-3 cells were cultured in McCoy's 5A medium containing 10% FBS and 1% penicillin-streptomycin in a 37 °C incubator with 5% CO_2_ under saturated humidity. SK-BR-3 cells were digested with 2.5% trypsin at passage. In addition, FBS supplemented with 10% DMSO was used to preserve cells at − 80 °C.

#### Cell uptake

SK-BR-3 cells were inoculated in a confocal dish and cultured for 12 h in an incubator containing 5% CO_2_ and saturated humidity at 37 °C. Then, the medium was removed, and FITC-labeled Ptb-Lip, Ptb-M-Lip and Ptb-M-lip-Her were added. The cells were incubated at 37 °C for 2 h and washed three times with PBS. The cells were then fixed with 4% paraformaldehyde for 15 min, permeabilized with 0.1% Triton for 10 min, and sealed with 1% BSA at 37 °C for 30 min. Finally, the cytoskeleton and nucleus were stained with phalloidin and Hoechst 33,342 at 37 °C, respectively, and cell uptake was observed by confocal laser scanning microscope (CLSM, BioTek Instruments, Winooski, VT).

Flow cytometry was used to assess cell uptake. SK-BR-3 cells were digested and inoculated in 6-well plates for 24 h. Then, the medium was removed, and FITC-labeled Ptb-Lip, Ptb-M-Lip and Ptb-M-Lip-Her were added for incubation for 2 h. Cell uptake was measured by flow cytometry (Agilent Biosciences Inc., Santa Clara, CA).

#### Homotypic targeting

To verify the homologous targeting of Ptb-M-Lip, we evaluated the uptake of Ptb-M-Lip in different cancer cells. SK-BR-3, A549, and C6 cells were seeded in confocal dishes at a density of 5 × 10^3^ and then incubated with Ptb-M-Lip for 2 h for cellular uptake as described above. Finally, the uptake of Ptb-M-Lip by different cancer cells was observed by CLSM.

#### Cytotoxicity analysis

To evaluate the toxicity of Ptb, Ptb-M-Lip and Ptb-M-Lip-Her on SK-BR-3 cells, an MTT assay was used to detect the inhibitory effect of Ptb on SK-BR-3 cell proliferation. After the cells were digested, cell counting plates were used to count the cells, and according to the counting results, the cell suspensions were diluted into cell suspensions with a concentration of 5 × 10^4^ cells/mL by medium. Cell suspensions (100 μL) were inoculated into 96-well plates and cultured for 24 h. After cell adhesion, Ptb, Ptb-M-Lip and Ptb-M-Lip-Her were diluted to different concentrations (equivalent concentrations of Ptb were 0.2, 0.4, 0.8, 1, 4, and 8 μg/mL), added to 96-well plates and incubated for 48 h. MTT (5 mg/mL) solution was added away from the light and incubated in the dark for 4 h. Then, the supernatant in the 96-well plate was discarded, and 100 μL DMSO was added and shaken in the dark at a low speed for 15 min. The optical density (OD) was measured and recorded at 492 nm using a microplate reader (VERSA max, Molecular Devices, Sunnyvale, CA) after the formazan was completely dissolved. The following formula was used to calculate cell viability:

$${\text{Cell}}\,{\text{viability}} = {\text{OD}}_{{\text{t}}} /{\text{OD}}_{{\text{c}}} \times 100\%$$

#### Live and dead cell staining

SK-BR-3 cells were inoculated into 24-well plates and cultured for 24 h. Ptb, Ptb-M-Lip and Ptb-M-Lip-Her (equivalent to 0.8 μg/mL Ptb) were added for 24 h. Calcein-AM/PI detection working solution (500 μL) was added to each well. Then, the cells were incubated in 37 °C darkness for 40 min, rinsed with PBS and observed under a fluorescence microscope (Leica, Wetzlar, Germany).

#### Flow cytometric detection of apoptosis

SK-BR-3 cells were digested and inoculated into 6-well plates at 1 × 10^5^ cells/well for 24 h. Then, Ptb, Ptb-M-Lip and Ptb-M-Lip-Her (equivalent to 0.8 µg/mL Ptb) were added to the 6-well plate for 48 h. The cells were digested with trypsin and then washed with PBS 3 times. The cells were resuspended in 500 μL binding buffer, added to 5 μL Annexin V-FITC and 7-AAD, and incubated for 15 min away from light. Finally, apoptosis was detected by flow cytometry (Agilent Biosciences Inc., Santa Clara, CA).

#### Detection of mitochondrial membrane potential

SK-BR-3 cells were inoculated into 12-well plates and cultured for 24 h. Then, Ptb, Ptb-M-Lip and Ptb-M-Lip-Her (equivalent to 0.8 µg/mL Ptb) were added for 24 h. JC-1 staining solution was added and incubated at 37 °C for 30 min in the dark. Finally, SK-BR-3 cells were observed with a fluorescence microscope (Leica, Wetzlar, Germany).

#### Immunofluorescence to detect apoptosis

SK-BR-3 cells were inoculated into 24-well plates and cultured for 24 h. Then, Ptb, Ptb-M-Lip and Ptb-M-Lip-Her (equivalent to 0.8 µg/mL Ptb) were added for 24 h. The cells were washed with PBS 3 times, fixed with 4% paraformaldehyde for 30 min, and drilled with 0.5% Triton for 15 min. The cells were then sealed with goat serum for 2 h and incubated at 4 °C overnight with anti-Caspase-3. The next day, the cells were incubated with the secondary antibody at room temperature for 2 h, and Hoechst 33,342 and phalloidin were added for 15 min. Finally, SK-BR-3 cells were observed under a fluorescence microscope (Leica, Wetzlar, Germany).

#### Western blot

Western blotting was used to detect the expression level of apoptotic proteins in SK-BR-3 cells. SK-BR-3 cells were first cultured in a culture flask, and then Ptb, Ptb-M-Lip and Ptb-M-Lip-Her (equivalent to 0.8 µg/mL Ptb) were added for 48 h. The cells were gently scraped from the flask over ice. Cells were collected and washed with PBS 3 times. RIPA lysis buffer was added to each sample and incubated on ice for 30 min. After centrifugation at 4 °C and 12,000 rpm for 30 min, the protein concentration in each sample was determined using a BCA protein assay kit. For western blot analysis, the protein (10 μL) in each sample was electrophoretically separated on a polyacrylamide gel and then transferred to a polyvinylidene fluoride (PVDF) film. The PVDF membrane was incubated in 1% BSA for 2 h and then incubated with primary antibodies (anti-Bcl-2, anti-Bax, and anti-Caspase-3) overnight at 4 °C. The next day, the secondary antibody was added and incubated at room temperature for 2 h. The membrane was treated with ECL chromogenic agent. Expression analysis was performed by Quantity One 1-D analysis software (Bio-Rad, Hercules, USA).

### In vivo experiment

#### Establishment of the mouse tumor model

BALB/c nude mice (females, 18–20 g) were purchased from Beijing Weitonglihua Experimental Animal Technology Co., Ltd. (Beijing, China). All mice were housed in the SPF Laboratory Animal Center of Jinzhou Medical University. The experiment was conducted in accordance with the Animal Management Regulations of Jinzhou Medical College. SK-BR-3 cells (5 × 10^6^) were injected into the adipose pad of the right mammary gland of nude mice. One week later, there was a mass in the fat pad of the right breast, indicating the successful establishment of the tumor model.

#### In vivo antitumor effect and safety

When the tumor volume reached 100 mm^3^, the nude mice were randomly divided into four groups: the control group, Ptb group, Ptb-M-Lip group and Ptb-M-Lip-Her group. Different preparations (equivalent to Ptb 10 mg/kg) were injected into the caudal vein, and the control group was given normal saline. The drug was administered every three days for a total of 7 doses. We measured the longest and shortest tumor diameters and mouse body weight before each administration. The tumor volume was calculated as follows:

$${\text{Volume}}\,{\text{of}}\,{\text{tumor}} = \left( {{\text{longest}}\,{\text{diameter}}} \right) \times \left( {{\text{shortest}}\,{\text{diameter}}} \right)^{2} /2.$$

The tumor inhibition rate was obtained according to the following formula:

$${\text{Tumor}}\,{\text{inhibition}}\,{\text{rate}} = (1 - {\text{W}}_{{\text{t}}} /{\text{W}}_{{\text{c}}} ) \times 100\%$$

where W_t_ is the mean weight of the tumor for each drug treatment group and W_c_ is the mean weight of the tumor for the control group.

The mice were killed after the last administration, and the tumor tissue and major organs (heart, liver, spleen, lung, and kidney) were removed. The tissues were fixed with 4% paraformaldehyde for 24 h, dehydrated and embedded in paraffin. Sections were stained with hematoxylin and eosin (H&E) and Ki-67 immunohistochemical staining. The sections were observed with a fluorescence microscope (Leica DMI 4000B, Wetzlar, Germany).

#### In vivo imaging

To further verify the targeting ability of Ptb-M-Lip-Her, FITC-labeled Ptb, Ptb-M-Lip, and Ptb-M-Lip-Her were injected into the tail vein of tumor-bearing nude mice. The mice were killed 3 h later, and the heart, liver, spleen, lung, kidney and tumor were removed. Fluorescence signal intensity in tumors and major organs was observed at 518 nm and 494 nm using an in vivo imaging system (IVIS Spectrum, PerkinElmer, Waltham, MA).

### Statistical analysis

The statistical analysis was performed using GraphPad Prism (version 8.0), and the results of all experiments were reported as the mean ± SD. When the *P* value was less than 0.05, it was considered statistically significant.

## Results and discussion

### Characterization of Ptb-M-Lip-Her

**Scheme 1:**
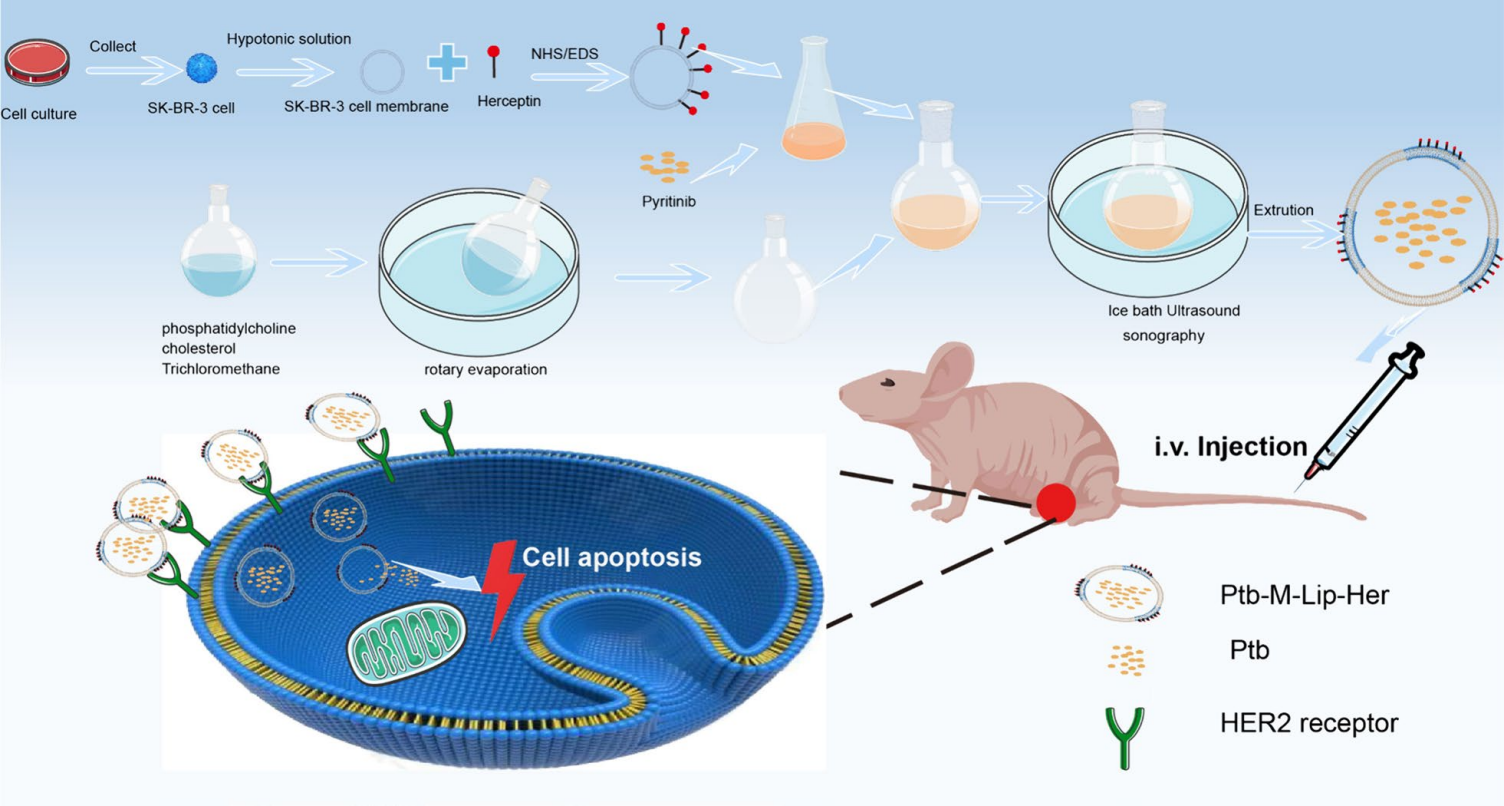
Schematic diagram of the Ptb-M-Lip-Her preparation process and its antitumor effect

**Fig. 1:**
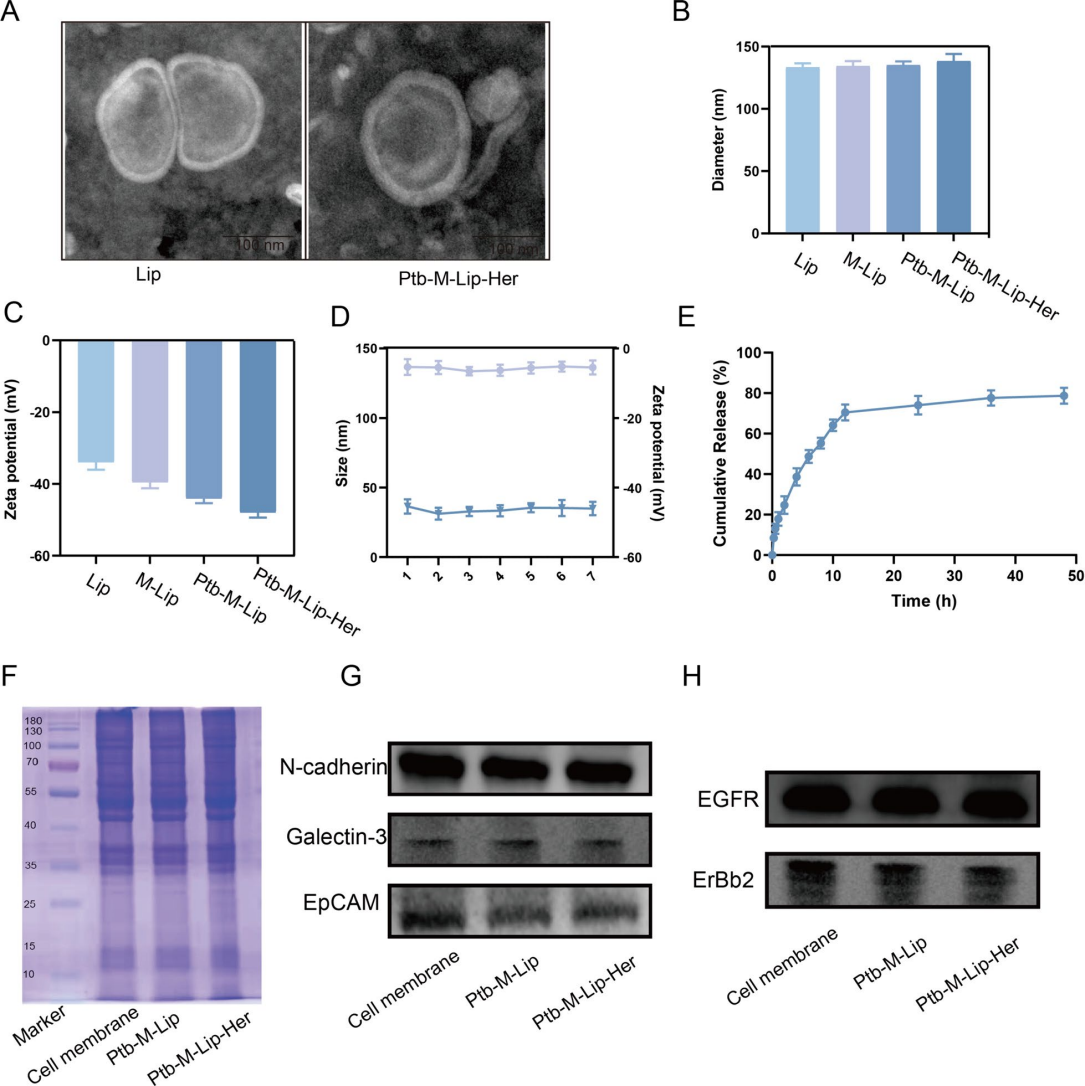
Characterization of Ptb-M-Lip-Her. **A** TEM images of Lip and Ptb-M-Lip-Her. **B** Particle size distribution of Lip, M-Lip, Ptb-M-Lip and Ptb-M-Lip-Her obtained by DLS. **C** Zeta potential of Lip, M-Lip, Ptb-M-Lip and Ptb-M-Lip-Her. **D** 7-day stability test of Ptb-M-Lip-Her. **E** Cumulative release curve of Ptb in Ptb-M-Lip-Her. **F** SDS-PAGE analysis of SK-BR-3 cell membrane, Ptb-M-Lip and Ptb-M-Lip-Her. **G** Western blot analysis (N-cadherin, Galectin-3, EpCAM) of SK-BR-3 cell membrane, Ptb-M-Lip and Ptb-M-Lip-Her. **H** Western blot analysis (EGFR, ErBb2) of SK-BR-3 cell membrane, Ptb-M-Lip and Ptb-M-Lip-Her. All data represent mean ± SD (n = 3)

The preparation process of Ptb-M-Lip-Her is shown in Scheme 1. The SK-BR-3 cell membrane was extracted from SK-BR-3 cells, and then Herceptin was connected to the surface of the cell membrane by amide bonds to obtain the functionalized SK-BR-3 cell membrane. Liposomes were prepared by thin film dispersion. The lipid membranes were hydrated with a saline solution containing Herceptin-modified cell membranes and pyrotinib. The system was then ultrasonicated in an ice bath and continuously extruded through a polycarbonate membrane to obtain Ptb-M-Lip-Her. The material was purified by a dextran gel column and administered to mice by caudal vein injection. Liposomes fused with cell membranes have homologous targeting and can actively target tumor tissues. At the same time, the HER2 receptor is highly expressed in tumor tissues. Herceptin, modified on the cell membrane surface, as a targeting ligand for HER2 receptors, further enhances the targeting and antitumor effects of Ptb-M-Lip-Her. As shown in Fig. 1A, the morphology and structure of Ptb-M-Lip-Her were observed by transmission electron microscopy (TEM). The structure of Ptb-M-Lip-Her was complete and spherical, with a particle size of approximately 140 nm. Compared with liposomes, the particle size of Ptb-M-Lip-Her is slightly increased. The results of TEM were further verified by the DLS method, and the particle size and zeta potential were determined (Fig. 1B, C). The particle size was 133.3 ± 2.2 nm for Lips, 134.3 ± 2.6 nm for M-Lips, 135.1 ± 1.6 nm for Ptb-M-Lip and 138.3 ± 3.2 nm for Ptb-M-Lip-Her, and the zeta potentials were − 33.9 ± 1.2 mV for Lips, − 39.6 ± 2.3 mV for M-Lip, − 44.1 ± 0.6 mV for Ptb-M-Lip and − 47.9 ± 2.0 mV for Ptb-M-Lip-Her. This demonstrated that the cell membrane admixture and drug loading did not significantly change the size of liposomes. In the stability analysis, the particle size and zeta potential of Ptb-M-Lip-Her did not change significantly within 7 days, demonstrating the stability of the nanosystem (Fig. 1D). As shown in Fig. 1E, the in vitro release experiment showed that the cumulative release rate of pyrotinib at 24 h of Ptb-M-Lip-Her was 74.02 ± 5.49%, which proved that the liposome modification of the fusion cell membrane regulated the drug release rate, making the drug release process slow and sustained. The systemic circulation time and bioavailability of Ptb-M-Lip-Her were further improved. SDS-PAGE analysis showed that the electrophoretic bands of Ptb-M-Lip and Ptb-M-Lip-Her were consistent with those of the SK-BR-3 cell membrane (Fig. 1F). This indicated that the liposome fused with the cell membrane successfully, and the composition of membrane surface proteins was not affected during the encapsulation process. In addition, we further verified this result by western blot experiments. The expression of adhesion molecules (e.g., N-cadherin, Galectin-3, and EpCAM) and labeled proteins (e.g., EGFR and ErBb2) on the SK-BR-3 cell membrane, Ptb-M-Lip and Ptb-M-Lip-Her surfaces were analyzed in Fig. 1G, H. According to the literature, these adhesion factors and proteins are highly expressed on the surface of tumor cell membranes [37]. The western blot results showed that the bioadhesion factors and labeled proteins on the SK-BR-3 cell membrane were also highly enriched in Ptb-M-Lip and Ptb-M-Lip-Her, which further proved the successful fusion between the cell membrane and liposomes.

### Targeting study of Ptb-M-Lip-Her

**Fig. 2:**
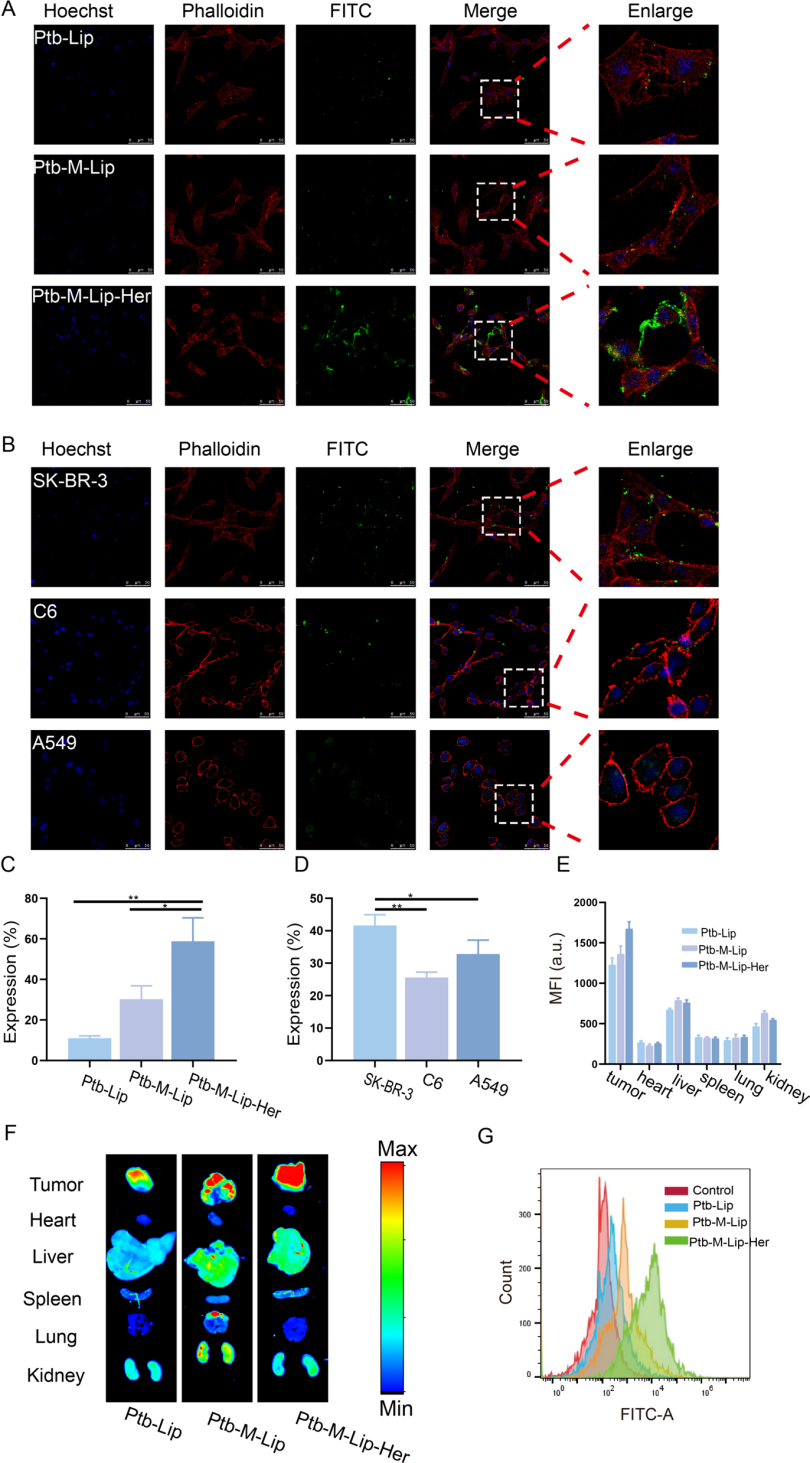
Targeting study. **A** CLSM images of FITC-labeled Ptb-Lip, Ptb-M-Lip and Ptb-M-Lip-Her uptaken by SK-BR-3 cells. Scale bars: 50 µm. **B** CLSM images of FITC-labeled Ptb-M-Lip uptaken by SK-BR-3 cells, C6 cells and A549 cells. Scale bars: 50 µm. **C** The fluorescence intensity of fluorescent images in A. **D** The fluorescence intensity of fluorescent images in B. **E** The fluorescence intensity in different organizations. **F** Tissue fluorescence imaging of FITC-labeled Ptb-Lip, Ptb-M-Lip and Ptb-M-Lip-Her. **G** Cellular uptake results by SK-BR-3 cells from flow cytometry. All data represented the mean ± SD (n = 3) (*p < 0.05; **p < 0.01; ***p < 0.001)

The targeting of Ptb-M-Lip-Her at the cellular level was verified by in vitro cell uptake assay and flow cytometry assay. FITC was used to label Ptb-Lip, Ptb-M-Lip and Ptb-M-Lip-Her and showed green fluorescence. The cell nucleus was stained with Hoechst 33,342 for blue fluorescence, and the cytoskeleton was stained with rhodamine phalloidin for red fluorescence. The uptake difference of Ptb-Lip, Ptb-M-Lip and Ptb-M-Lip-Her by SK-BR-3 cells was observed by fluorescence microscopy. As shown in Fig. 2A, C, SK-BR-3 cells had significantly higher uptake of Ptb-M-Lip than Ptb-Lip and further enhanced uptake of Ptb-M-Lip-Her compared with Ptb-M-Lip and Ptb-Lip. These results indicated that the targeted delivery of the drug was enhanced after the fusion of the SK-BR-3 cell membrane with liposomes, and the modification of the cell membrane surface with Herceptin further improved the targeting ability of Ptb-M-Lip-Her. To further prove the homologous targeting of the SK-BR-3 cell membrane, the uptake of Ptb-M-Lip by SK-BR-3 cells, C6 cells and A549 cells was observed by CLSM. As shown in Fig. 2B, D, the green fluorescence was weak in C6 cells and A549 cells and the strongest in SK-BR-3 cells. This showed that SK-BR-3 cells had the strongest uptake of Ptb-M-Lip. These results proved that the targeting of Ptb-M-Lip can be improved due to the homologous targeting ability of the SK-BR-3 cell membrane after the fusion of liposomes with the SK-BR-3 cell membrane. The uptake of Ptb-Lip, Ptb-M-Lip, and Ptb-M-Lip-Her by SK-BR-3 cells was measured by flow cytometry, and the results were consistent with those observed by CLSM (Fig. 2G). The cell uptake of Ptb-Lip, Ptb-M-Lip and Ptb-M-Lip-Her was 13.2 ± 2.21%, 38.4 ± 4.23% and 89.6 ± 3.01%, respectively (Fig. 1S). The cell uptake rate of Ptb-M-Lip-Her was significantly higher than that of Ptb-Lip and Ptb-M-Lip. This further demonstrated that Ptb-M-Lip-Her had an outstanding ability to target SK-BR-3 cells. In vivo imaging studies further validated the targeting of Ptb-M-Lip-Her at the animal level. FITC-labeled Ptb-Lip, Ptb-M-Lip, and Ptb-M-Lip-Her were injected into nude mice through the tail vein. The fluorescence intensity of tumors and major organs (heart, liver, spleen, lung and kidney) was then observed using a small animal imaging system (Fig. 2E, F). The fluorescence intensity of tumor sites in the Ptb-M-Lip group was higher than that in the Ptb-Lip group, and the fluorescence intensity of tumor sites in the Ptb-M-Lip-Her group was the strongest. These results showed that the enrichment degree of the Ptb-M-Lip-Her group was the highest at the tumor site. These results indicated that Ptb-M-Lip-Her has great potential in the targeted treatment of breast cancer.

### In vitro apoptosis and toxicity analysis

**Fig. 3:**
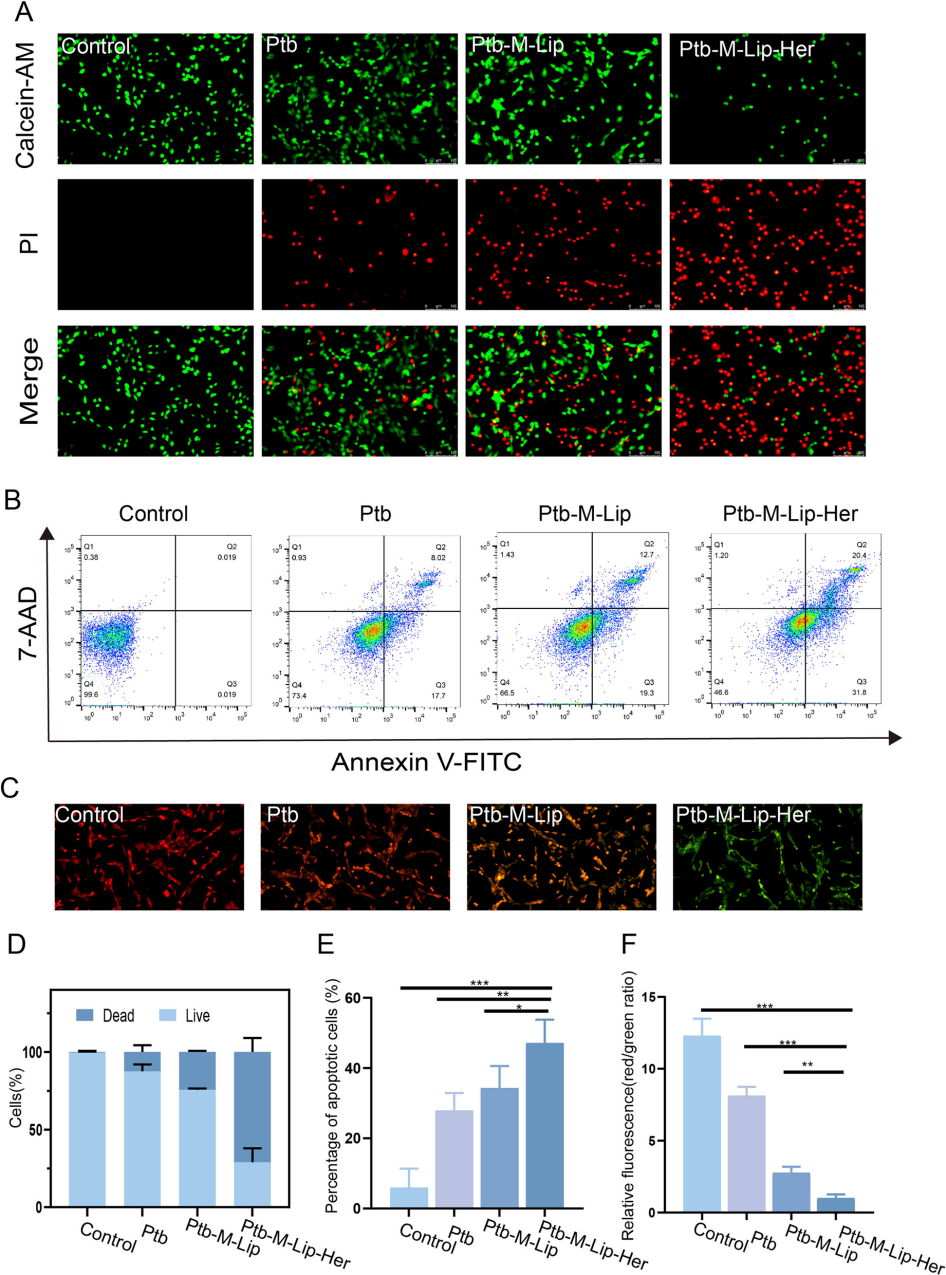
In vitro apoptosis and toxicity analysis. **A** Fluorescence images of live and dead cells stained with Calcein-AM/PI after different treatments. Green fluorescence indicates live cells, red fluorescence indicates dead cells. Scale bars: 50 µm. **B** Flow cytometry analysis of apoptosis ratio. **C** Fluorescence image of SK-BR-3 cells stained with JC-1 after different treatments. Scale bars: 50 µm. **D** Statistical analysis of the ratio of live and dead cells in A. **E** Statistical analysis of apoptosis rate in B. **F** The ratio changes of red fluorescence intensity to green fluorescence intensity in C. All data represented the mean ± SD (n = 3) (*p < 0.05; **p < 0.01; ***p < 0.001)

**Fig. 4:**
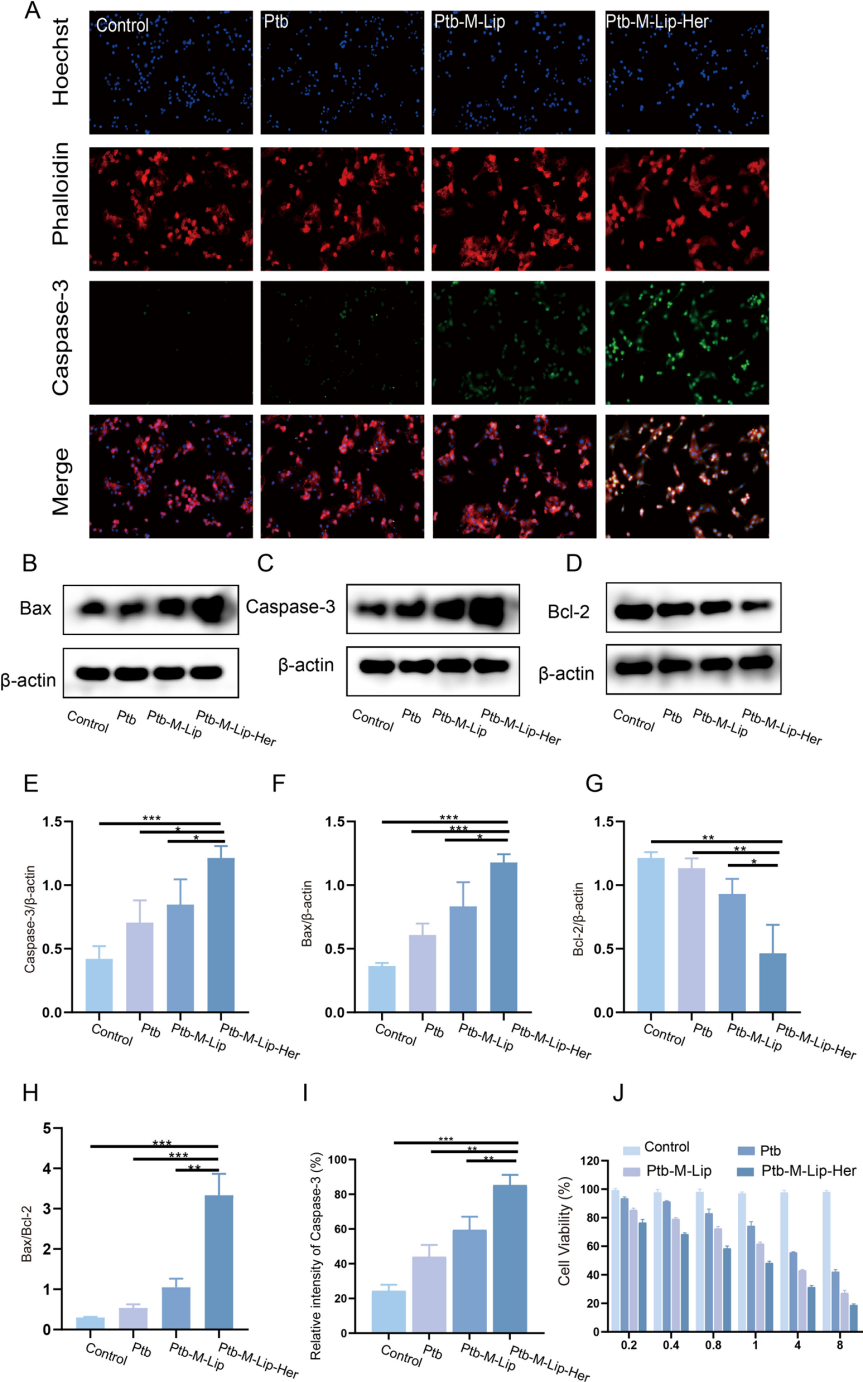
In vitro apoptosis and toxicity analysis. **A** Caspase-3 immunofluorescence images of SK-BR-3 cells after different treatments. Scale bars: 50 µm. **B**–**D** Western blotting was used to detect the expression of cytokines (Bax, Caspase-3, Bcl-2) in different groups. **E**–**H** Quantitative analysis of the expression levels of Caspase-3, Bax, Bcl-2 and Bax/Bcl-2. **I** Relative fluorescence intensity analysis of Caspase-3. **J** The cell viability of SK-BR-3 cells after incubated with Ptb, Ptb-M-Lip and Ptb-M-Lip-Her. All data represented the mean ± SD (n = 3) (*p < 0.05; **p < 0.01; ***p < 0.001)

To investigate the proapoptotic effect of Ptb-M-Lip-Her, immunofluorescence, flow cytometry and western blot tests were performed in vitro. The immunofluorescence experiments included Calcein-AM/PI staining, mitochondrial membrane potential determination and Caspase-3 immunofluorescence. A Calcein-AM/PI double staining kit was used to stain living and dead cells to verify the cytotoxicity of SK-BR-3 cells induced by different nanoparticles. Calcein-AM and PI can stain live cells green and dead cells red. As shown in Fig. 3A, D, all cells in the control group showed green fluorescence, indicating that SK-BR-3 cells were in the proliferative stage. The red fluorescence of the Ptb-M-Lip group was higher than that of the Ptb group, indicating that Ptb-M-Lip had stronger toxicity to SK-BR-3 cells. The red fluorescence was significantly increased and the green fluorescence was decreased in the Ptb-M-Lip-Her group, which indicated that the cells were mostly apoptotic. This proved that Ptb-M-Lip-Her had the most pronounced proapoptotic effect on SK-BR-3 cells. Mitochondrial membrane potential reduction is a landmark event of early apoptosis. JC-1 is an ideal fluorescent probe for the detection of mitochondrial membrane potential. When the mitochondrial membrane potential was high, JC-1 aggregated in the mitochondrial matrix to form a polymer, showing red fluorescence. When the mitochondrial membrane potential decreased, JC-1 could not accumulate in the mitochondrial matrix and produced green fluorescence. The apoptosis rate can be measured by examining the ratio of red fluorescence to green fluorescence. The lower the ratio, the stronger the ability to promote apoptosis of tumor cells. As shown in Fig. 3C, F, the ratio of red fluorescence to green fluorescence in the Ptb, Ptb-M-Lip and Ptb-M-Lip-Her groups gradually decreased after administration, indicating that the ability to promote apoptosis of SK-BR-3 cells was gradually enhanced. Among them, the red and green fluorescence ratio of the Ptb-M-Lip-Her group was the lowest, indicating that Ptb-M-Lip-Her had the most toxic effect on SK-BR-3 cells. Then, Caspase-3 immunofluorescence was used to further detect apoptosis. As shown in Fig. 4A, I, the green fluorescence intensity gradually increased after treatment with Ptb, Ptb-M-Lip and Ptb-M-Lip-Her. The results showed that Ptb-M-Lip-Her had the strongest apoptotic effect on SK-BR-3 cells. Flow cytometry was used to further quantitatively analyze the proapoptotic effect of Ptb-M-Lip-Her (Fig. 3B, E). The apoptosis rate of the Ptb-M-Lip-Her group was 47.12 ± 2.28%, while that of the Ptb group and Ptb-M-Lip group were 27.93 ± 1.56% and 34.31 ± 1.49%, respectively. The apoptosis rate of the Ptb-M-Lip-Her group was significantly higher than that of the other groups, suggesting that the Ptb-M-Lip-Her group could significantly promote the apoptosis of SK-BR-3 cells. The expression levels of Caspase 3, Bcl-2 and Bax in tumor cells after administration were detected by western blot, which further confirmed the above conclusions. After SK-BR-3 cells were treated with Ptb, Ptb-M-Lip and Ptb-M-Lip-Her, the expression of pro-apoptotic proteins (Caspase 3 and Bax) was gradually increased, while the expression of anti-apoptotic protein (Bcl-2) was gradually decreased (Fig. 4B–G). The ratio of Bax/Bcl-2 in the Ptb-M-Lip-Her group was the highest (Fig. 4H). These results further proved that Ptb-M-Lip-Her had the most obvious apoptotic effect on SK-BR-3 cells. The toxicity of Ptb, Ptb-M-Lip and Ptb-M-Lip-Her to SK-BR-3 cells was detected by MTT assay (Fig. 4J). When the concentration of Ptb was 1 μg/mL, the survival rate of Ptb-M-Lip-Her cells was 48.32 ± 1.29%, which was significantly lower than that of Ptb (74.30 ± 1.62%) and Ptb-M-Lip (61.77 ± 1.43%). When the concentration of Ptb reached 4 μg/mL, the cell viabilities of Ptb, Ptb-M-Lip and Ptb-M-Lip-Her were 55.69 ± 1.96%, 43.00 ± 1.23% and 31.43 ± 1.89%, respectively. After treatment with Ptb-M-Lip-Her, the cell viability decreased with increasing Ptb concentration, and Ptb-M-Lip-Her showed the strongest inhibitory effect on SK-BR-3 cells. The IC50 values of Ptb, Ptb-M-Lip and Ptb-M-Lip-Her were 5.04 ± 15.31 μg/mL, 3.36 ± 6.15 μg/mL and 2.67 ± 3.16 μg/mL, respectively. The IC50 results further proved that Ptb-M-Lip-Her had a good antitumor effect.

### In vivo antitumor effect and safety

**Fig. 5:**
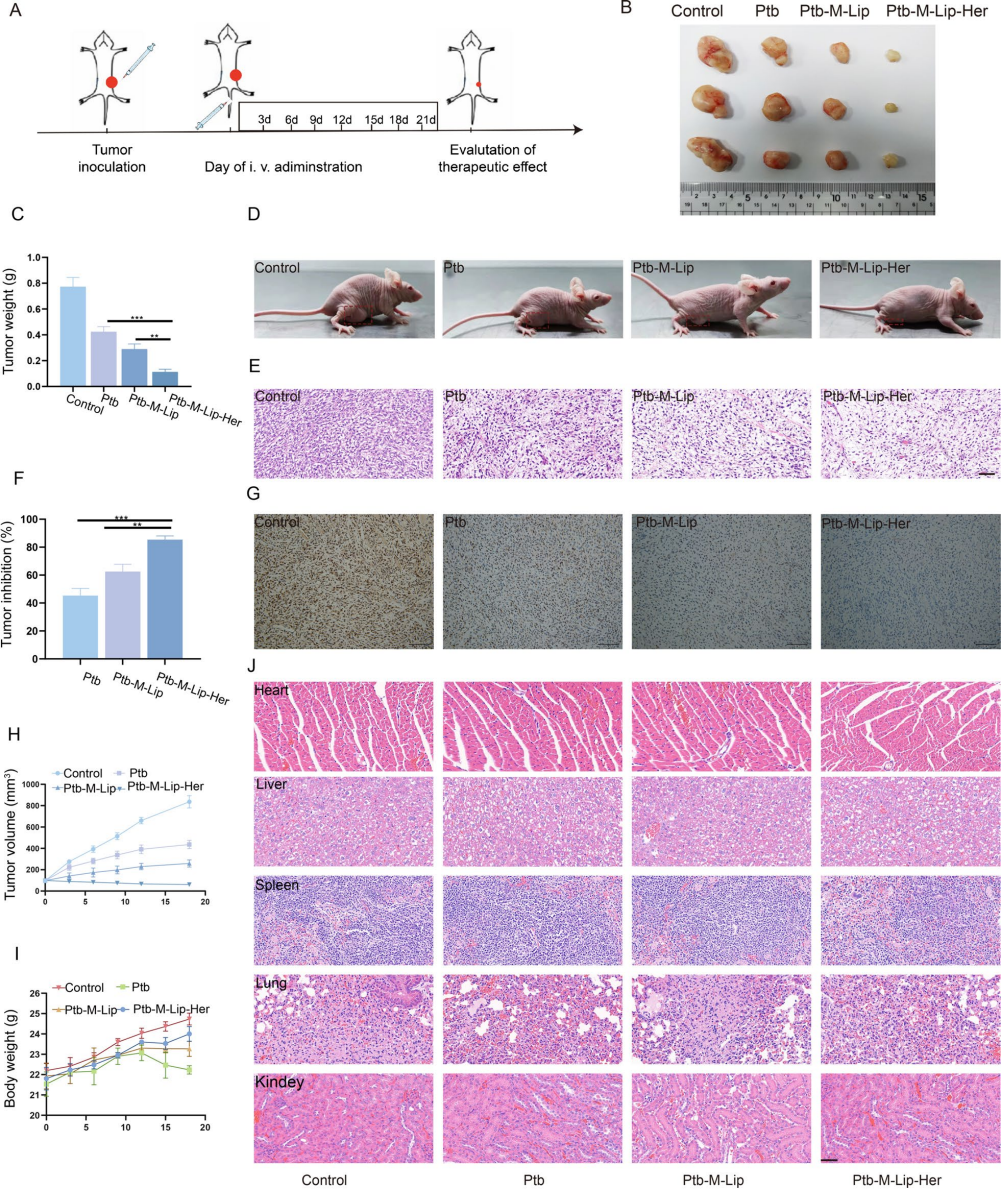
In vivo antitumor effect and safety. **A** Tumor model establishment and drug administration time diagram. **B** Tumor images after 21 days of treatment in different groups. **C** Tumor weight after 21 days of treatment in different groups. **D** Photographs of tumor-bearing mice. **E** Tumor H&E staining in different treatment groups. Scale bars: 100 µm. **F** Tumor inhibition rate after 21 days of treatment in different groups. **G** Tumor Ki-67 immunohistochemical imaging in different treatment groups. Scale bars: 100 µm. **H** The change curve of tumor volume of tumor-bearing mice during 18 days of treatment. **I** The weight change curve of tumor-bearing mice during 18 days of treatment. **J** H&E staining of the main organs in different treatment groups. Scale bars: 100 µm. All data represented the mean ± SD (n = 3) (*p < 0.05; **p < 0.01; ***p < 0.001)

After 21 days of administration, tumor tissue was removed and photographed. As shown in Fig. 5B, we observed that the tumor size of the Ptb-M-Lip-Her group was significantly smaller than that of the other groups. In Fig. 5C, the tumor weight of the Ptb-M-Lip-Her group was 113.33 ± 4.21 mg, which was significantly lower than that of the Ptb and Ptb-M-Lip groups (423.33 ± 9.48 mg and 290.01 ± 8.43 mg). The tumor inhibition rates of Ptb and Ptb-M-Lip were 45.26 ± 6.81% and 62.50 ± 5.03%, respectively, while that of the Ptb-M-Lip-Her group was 85.34 ± 3.42% (Fig. 5F). Tumor volumes in the Ptb, Ptb-M-Lip and Ptb-M-Lip-Her groups were 436 ± 45.25 mm^3^, 259 ± 31.27 mm^3^ and 61.33 ± 53.02 mm^3^, respectively, and the tumor volumes in the Ptb-M-Lip-Her group were significantly lower than those in the other groups (Fig. 5H). These results clearly indicated that Ptb-M-Lip-Her could significantly inhibit tumor growth. The body weight changes of mice after treatment are shown in Fig. 5I. The body weight of mice in the Ptb group decreased gradually with the extension of administration time, indicating that the toxic side effects in the Ptb group were obvious. However, the weights of the Ptb-M-Lip and Ptb-M-Lip-Her groups continued to increase, and the weight of the Ptb-M-Lip-Her group was the most significant. These results indicated that the active targeting ability of Ptb-M-Lip-Her significantly reduced the side effects of pyrotinib. To further demonstrate the antitumor effect of Ptb-M-Lip-Her, H&E staining and Ki67 staining were performed on tumor sections (Fig. 5E, G). In tumor H&E staining, the Ptb-M-Lip-Her group showed obvious necrosis compared with the Ptb-M-Lip and Ptb groups. Ki67 immunohistochemical staining showed that the brown nuclei of proliferating cells in the Ptb-M-Lip-Her group were significantly fewer than those in the other groups. These results all confirmed that Ptb-M-Lip-Her has obvious antitumor activity in vivo. No significant pathological changes were observed in H&E staining images of major organs (heart, liver, spleen, lung, and kidney) of mice after different treatments, indicating that Ptb-M-Lip-Her had good biological safety (Fig. 5J). In conclusion, Ptb-M-Lip-Her has great prospects in the treatment of HER2-positive breast cancer.

## Conclusion

The bionic nanosystem of trastuzumab-functionalized SK-BR-3 cell membrane hybrid liposome-coated pyrotinib (Ptb-M-Lip-Her) was successfully prepared for the treatment of HER2-positive breast cancer. In vitro cell experiments showed that Ptb-M-Lip-Her had a good targeting effect and promoted apoptosis of SK-BR-3 cells. In vivo animal experiments further demonstrated that Ptb-M-Lip-Her could accumulate in large quantities in tumors and had an obvious inhibitory effect on tumor growth. In conclusion, Ptb-M-Lip-Her has important application prospects in the targeted therapy of HER2-positive breast cancer.

### Supplementary Information

Supplementary Material 1.

## Data Availability

The raw/processed data required to reproduce these findings cannot be shared at this time as the data also forms part of an ongoing study.

## References

[1] Kunte S, Abraham J, Montero AJ (2020). Novel HER2-targeted therapies for HER2-positive metastatic breast cancer. Cancer.

[2] Waks AG, Winer EP (2019). Breast cancer treatment: a review. JAMA.

[3] Loibl S, Poortmans P, Morrow M, Denkert C, Curigliano G. Breast cancer [published correction appears in Lancet. 2021 May 8;397(10286):1710]. Lancet. 2021;397(10286):1750–1769. 10.1016/S0140-6736(20)32381-3 10.1016/S0140-6736(20)32381-3 33812473

[4] Pallerla S, Abdul AURM, Comeau J, Jois S (2021). Cancer vaccines, treatment of the future: with emphasis on HER2-positive breast cancer. Int J Mol Sci.

[5] Harbeck N (2022). Neoadjuvant and adjuvant treatment of patients with HER2-positive early breast cancer. Breast.

[6] Jacob SA, Do V, Wilson BE, Ng WL, Barton MB (2021). The value of first-line chemotherapy and targeted therapy in the treatment of breast cancer. Eur J Cancer Care (Engl).

[7] Liyanage PY, Hettiarachchi SD, Zhou Y (2019). Nanoparticle-mediated targeted drug delivery for breast cancer treatment. Biochim Biophys Acta Rev Cancer.

[8] Lamtha T, Tabtimmai L, Bangphoomi K (2021). Generation of a nanobody against HER2 tyrosine kinase using phage display library screening for HER2-positive breast cancer therapy development. Protein Eng Des Sel.

[9] Zheng G, Guo Z, Li W (2021). Interaction between HLA-G and NK cell receptor KIR2DL4 orchestrates HER2-positive breast cancer resistance to trastuzumab. Signal Transduct Target Ther.

[10] Dormann C (2020). Metastatic human epidermal growth factor receptor 2-positive breast cancer: current treatment standards and future perspectives. Breast Care (Basel).

[11] Abu Samaan TM, Samec M, Liskova A, Kubatka P, Büsselberg D (2019). Paclitaxel's mechanistic and clinical effects on breast cancer. Biomolecules.

[12] Oh DY, Bang YJ (2020). HER2-targeted therapies—a role beyond breast cancer. Nat Rev Clin Oncol.

[13] Zheng X, Zhao Y, Jia Y (2021). Biomimetic co-assembled nanodrug of doxorubicin and berberine suppresses chemotherapy-exacerbated breast cancer metastasis. Biomaterials.

[14] Xuhong JC, Qi XW, Zhang Y, Jiang J (2019). Mechanism, safety and efficacy of three tyrosine kinase inhibitors lapatinib, neratinib and pyrotinib in HER2-positive breast cancer. Am J Cancer Res.

[15] Iancu G, Serban D, Badiu CD (2022). Tyrosine kinase inhibitors in breast cancer (review). Exp Ther Med.

[16] Xuhong J, Qi X, Tang P (2020). Neoadjuvant Pyrotinib plus trastuzumab and chemotherapy for stage I-III HER2-positive breast cancer: a phase II clinical trial. Oncologist.

[17] Schlam I, Swain SM (2021). HER2-positive breast cancer and tyrosine kinase inhibitors: the time is now. NPJ Breast Cancer.

[18] Li Q, Wang Y, Zhu M, Gu Y, Tang Y (2021). Clinical observation of neoadjuvant chemotherapy with pyrotinib plus trastuzumab in HER2-positive breast cancer: a cohort study. Gland Surg.

[19] Ai X, Song Z, Jian H (2021). Pyrotinib combined with thalidomide in advanced non-small-cell lung cancer patients harboring HER2 exon 20 insertions (PRIDE): protocol of an open-label, single-arm phase II trial. BMC Cancer.

[20] Fang C, Wen J, Kang M, Zhang Y, Chen Q, Ren L (2022). Incidence and management of pyrotinib-associated diarrhea in HER2-positive advanced breast cancer patients. Ann Palliat Med.

[21] Kusumastuti R, Kumagai Y, Ishihara S (2022). Mammaglobin 1 mediates progression of trastuzumab-resistant breast cancer cells through regulation of cyclins and NF-κB. FEBS Open Bio.

[22] Kumar S, Das S, Sun J (2022). Mixed lineage kinase 3 and CD70 cooperation sensitize trastuzumab-resistant HER2+ breast cancer by ceramide-loaded nanoparticles. Proc Natl Acad Sci USA.

[23] Zhao Z, Ma X, Zhang R (2021). A novel liposome-polymer hybrid nanoparticles delivering a multi-epitope self-replication DNA vaccine and its preliminary immune evaluation in experimental animals. Nanomedicine.

[24] Hamelmann NM, Paats JD, Paulusse JMJ (2021). Cytosolic delivery of single-chain polymer nanoparticles. ACS Macro Lett.

[25] Du J, Zong L, Li M (2022). Two-pronged anti-tumor therapy by a new polymer-paclitaxel conjugate micelle with an anti-multidrug resistance effect. Int J Nanomed.

[26] Ghosh B, Biswas S (2021). Polymeric micelles in cancer therapy: state of the art. J Control Release.

[27] Chen F, Li Y, Lin X, Qiu H, Yin S (2021). Polymeric systems containing supramolecular coordination complexes for drug delivery. Polymers (Basel).

[28] Costa D, Santo D, Domingues C, Veiga F, Faneca H, Figueiras A (2021). Recent advances in peptide-targeted micelleplexes: current developments and future perspectives. Int J Pharm.

[29] Zhu Y, Liang J, Gao C (2021). Multifunctional ginsenoside Rg3-based liposomes for glioma targeting therapy. J Control Release.

[30] Moghassemi S, Dadashzadeh A, Azevedo RB, Feron O, Amorim CA (2021). Photodynamic cancer therapy using liposomes as an advanced vesicular photosensitizer delivery system. J Control Release.

[31] Wang J, Zhu M, Nie G (2021). Biomembrane-based nanostructures for cancer targeting and therapy: From synthetic liposomes to natural biomembranes and membrane-vesicles. Adv Drug Deliv Rev.

[32] Chiappisi L, Hoffmann I, Gradzielski M (2022). Membrane stiffening in Chitosan mediated multilamellar vesicles of alkyl ether carboxylates. J Colloid Interface Sci.

[33] Ye H, Hu X, Wen Y (2022). Exosomes in the tumor microenvironment of sarcoma: from biological functions to clinical applications. J Nanobiotechnol.

[34] Chen Z, Zhao P, Luo Z (2016). Cancer cell membrane-biomimetic nanoparticles for homologous-targeting dual-modal imaging and photothermal therapy. ACS Nano.

[35] Fan Y, Hao W, Cui Y (2021). Cancer cell membrane-coated nanosuspensions for enhanced chemotherapeutic treatment of glioma. Molecules.

[36] An J, Jiang X, Wang Z (2022). Codelivery of minocycline hydrochloride and dextran sulfate via bionic liposomes for the treatment of spinal cord injury. Int J Pharm.

[37] Harjunpää H, Llort Asens M, Guenther C, Fagerholm SC (2019). Cell adhesion molecules and their roles and regulation in the immune and tumor microenvironment. Front Immunol.

