# Supplementary material for: Trastuzumab-functionalized bionic pyrotinib liposomes for targeted therapy of HER2-positive breast cancer
Source: Breast Cancer Res. 2024 Jun 12;26:99. doi: 10.1186/s13058-024-01853-2 (PMC11167944; doi:10.1186/s13058-024-01853-2)
Supplement: Supplementary file 1 — Supplementary Material 1. [file 13058_2024_1853_MOESM1_ESM.docx]

Supporting Information

Jiaqun Du ^b^, Xiaobang Liu ^b^, Junpeng Sun ^b^, Qian Wu ^b^, Yu Hu ^b^, Huan Shi ^b^, Li Zheng ^b^, Ying Liu^b,*^, Chao Wu^b,*^ and Yu Gao^a,*^

^a^ Department of Medical Oncology, the First Affiliated Hospital of Jinzhou Medical University, No.2, the Fifth Section of Renmin Street, Guta District, Jinzhou, Liaoning Province 121001, China

^b^ Pharmacy School, Jinzhou Medical University, 40 Songpo Road, Linghe, Jinzhou, Liaoning, 121001, China

Corresponding author:

Dr. Yu Gao

E-mail: gaoy1@jzmu.edu.cn

Addrress: Department of Medical Oncology, the First Affiliated Hospital of Jinzhou Medical University, No.2, the Fifth Section of Renmin Street, Guta District, Jinzhou, Liaoning Province 121001, China

Dr. Chao Wu,

E-mail: [wuchao@jzmu.edu.cn](mailto:wuchao@jzmu.edu.cn)

Addrress: Pharmacy School, Jinzhou Medical University, 40 Songpo Road, Linghe, Jinzhou, Liaoning, 121001, China

Dr.Ying Liu

E-mail: [ly0924_1979@163.com](mailto:ly0924_1979@163.com)

Addrress: Pharmacy School, Jinzhou Medical University, 40 Songpo Road, Linghe, Jinzhou, Liaoning, 121001, China

Author E-mail address:

Jiaqun Du: [djq1808@163.com](mailto:djq1808@163.com)

Xiaobang Liu: [lxb1025431521@163.com](mailto:lxb1025431521@163.com)

Junpeng Sun: sjp783686581@163.com

Qian Wu: [wuqian_0103@163.com](mailto:wuqian_0103@163.com)

Yu Hu: huyu980908@163.com

Huan Shi: [17393387208@163.com](mailto:17393387208@163.com)

Li Zheng: zhengl@jzmu.edu.cn

**Fig. S1.** Analysis of cell uptake results of SK-BR-3 cells in flow cytometry.
